# NLRP3 Inflammasome: A Promising Therapeutic Target for Drug-Induced Toxicity

**DOI:** 10.3389/fcell.2021.634607

**Published:** 2021-04-12

**Authors:** Shanshan Wei, Wanjun Ma, Bikui Zhang, Wenqun Li

**Affiliations:** ^1^Department of Pharmacy, The Second Xiangya Hospital, Central South University, Changsha, China; ^2^Institute of Clinical Pharmacy, Central South University, Changsha, China

**Keywords:** drug-induced toxicity, NLRP3 inflammasome, IL-1β, hepatotoxicity, nephrotoxicity, cardiotoxicity

## Abstract

Drug-induced toxicity, which impairs human organ function, is a serious problem during drug development that hinders the clinical use of many marketed drugs, and the underlying mechanisms are complicated. As a sensor of infections and external stimuli, nucleotide-binding oligomerization domain (NOD)-like receptor family pyrin domain containing 3 (NLRP3) inflammasome plays a key role in the pathological process of various diseases. In this review, we specifically focused on the role of NLRP3 inflammasome in drug-induced diverse organ toxicities, especially the hepatotoxicity, nephrotoxicity, and cardiotoxicity. NLRP3 inflammasome is involved in the initiation and deterioration of drug-induced toxicity through multiple signaling pathways. Therapeutic strategies via inhibiting NLRP3 inflammasome for drug-induced toxicity have made significant progress, especially in the protective effects of the phytochemicals. Growing evidence collected in this review indicates that NLRP3 is a promising therapeutic target for drug-induced toxicity.

## Introduction

Drugs can regulate physiological function and be used in the prevention, treatment, and diagnosis of diseases. However, drugs may also result in toxic reactions, characterized by physiological abnormalities or even pathological changes in organs structure. Therefore, drug-induced toxicity is a vital problem in drug development and clinical drug application (Björnsson, 2016; Onakpoya et al., 2016). The use of many common drugs is limited by drug-induced organ injury, such as statins (Bouitbir et al., 2020), rifampicin (Chen et al., 2017), anthracyclines (Avila et al., 2018), and cisplatin (Klockl et al., 2020). Moreover, some drugs considered very safe can cause organ damage when they are taken in too high a dose or when idiosyncratic reaction occurs (Larson et al., 2005; Matsuo et al., 2014). Abundant evidence suggested that mitochondrial injury, innate immune response, inflammation, and cell death are responsible for the occurrence and pathogenesis of most drug-induced toxicity (Uetrecht, 2019). However, detailed molecular mechanisms remain elusive.

Inflammasomes are a group of cytosolic multimeric protein complexes that serve as a sensor of innate immunity in response to invading pathogen-associated molecular patterns (PAMPs) or endogenous danger-associated molecular patterns (DAMPs) (Martinon et al., 2002). Upon activation, inflammasomes trigger the cleavage and release of proinflammatory cytokines interleukin 1β (IL-1β) and interleukin 18 (IL-18) (Bauernfeind et al., 2009). To date, several subtypes of inflammasomes have been identified (de Zoete et al., 2014), among which the nucleotide-binding oligomerization domain (NOD)-like receptor family pyrin domain containing 3 (NLRP3) inflammasome is the best characterized and most studied one. NLRP3 inflammasome is an intracellular protein complex that initiates cellular injury via assembly of NLRP3, apoptosis-associated speck-like protein (ASC), and pro-caspase-1 in response to microbial infection and sterile stressors. Aberrant activation of NLRP3 has been implicated in the pathogenesis of various autoimmune, chronic inflammatory, and metabolic diseases such as Cryopyrin-associated periodic syndrome, atherosclerosis, obesity, Alzheimer’s disease, and Parkinson’s disease (Rheinheimer et al., 2017; Heneka et al., 2018; Booshehri and Hoffman, 2019; Chen S. W. et al., 2020). Several studies point out that NLRP3 inflammasome plays a pivotal role in the occurrence of organ damage induced by environmental toxicants (Zhou et al., 2019) and pesticides (Gao et al., 2019), as well as drugs applied in the clinic (Jaeschke and Ramachandran, 2020). For example, NLRP3 inflammasome activation initiated by death-associated protein kinase (DAPK) or mitochondrial reactive oxygen species (mtROS) played a key role in causing acute lung injury, acute kidney injury, and long-term cognitive impairment due to paraquat exposure (Chen et al., 2015; Liu et al., 2015, 2017).

Herein, we summarize the current literature regarding the potential role of NLRP3 inflammasome in the pathogenesis of drug-induced toxicity and evaluate the contribution of NLRP3 inflammasome activation in the progression of organ damage. We also found that many phytochemicals, as well as a few molecules, showed obvious alleviating effects on drug-induced toxicity by inhibiting NLRP3 inflammasome (Kim and Park, 2018; Wang S. et al., 2019; Yang et al., 2020; Zou et al., 2020). Recent research progress about the protection effects of phytochemicals is highlighted in this review.

## NLRP3 Inflammasome: A Brief Overview

### Structure of NLRP3 Inflammasome

The NLRP3 inflammasome comprises the sensor protein NLRP3, the adaptor protein ASC, and the downstream effector pro-caspase-1 (Srinivasula et al., 2002). NLRP3 is the best characterized cytosolic nod-like pattern recognition receptor and a tripartite protein that contains a central nucleotide-binding oligomerization (NACHT) domain, an amino-terminal pyrin domain (PYD), and a carboxy-terminal leucine-rich repeat (LRR) domain. The NACHT domain has ATPase activity that is vital for NLRP3 self-association and function (Duncan et al., 2007). ASC is a two-domain protein consisting of an N-terminal PYD and a C-terminal caspase activation and recruitment domain (CARD; Lu et al., 2014). The pro-caspase-1 is an inactive 45 kDa precursor protein in the resting state (Ayala et al., 1994). Danger signals are sensed by the NLRP3 LRR domain, which leads to the oligomerization of NLRP3 monomers through their NACHT domains. Upon activation, the NLRP3 protein interacts with ASC via PYD, and the CARD domain of ASC recruits pro-caspase-1 through interaction with its CARD domain, and enables proximity-induced caspase 1 self-cleavage and activation (Boucher et al., 2018). Apart from CARD, pro-caspase 1 contains a central large catalytic domain (p20) and a carboxy-terminal small catalytic subunit domain (p10), which play key roles in activating the downstream cytokines or gasdermind D (GSDMD) signaling pathway (Boucher et al., 2018).

### Priming and Activation of NLRP3 Inflammasome

Canonical activation pathway of NLRP3 inflammasome requires two parallel and independent steps: priming (transcription) and activation (oligomerization). The priming step allows for transcription upregulation of the nuclear factor-kappa B (NF-κB)-mediated NLRP3, pro-IL-1β, and pro-IL-18 genes expressions (Franchi et al., 2009). This transcriptional upregulation can be induced through the recognition of various PAMPs or DAMPs that engage pattern-recognition receptors (PRRs), such as Toll-like receptor 4 (TLR4) or nucleotide-binding oligomerization domain-containing protein 2 (NOD2), or through identifying cytokines such as tumor necrosis factor (TNF) and IL-1β (Bauernfeind et al., 2009; Xing et al., 2017).

After priming, NLRP3 inflammasome activation can occur in response to an array of pathogens or endogenous DAMPs, which induces the oligomerization of NLRP3 and the recruitment of the ASC and pro-caspase1. Multiple upstream signals including efflux of potassium ions (K^+^) or chloride ions (Cl^–^), flux of calcium ions (Ca^2+^), metabolic changes, and trans-Golgi disassembly are suggested to be responsible for the NLRP3 inflammasome activation (Heid et al., 2013; Swanson et al., 2019). After ATP stimulation, P2X7 promotes Ca^2+^ and Na^+^ influx and coordinates with the K^+^ channel TWIK2 (two-pore domain weakly inwardly rectifying K^+^ channel 2), which mediates K^+^ efflux (Di et al., 2018). Furthermore, K^+^ efflux can regulate Ca^2+^ flux by acting as a counter ion at the plasma membrane for Ca^2+^ influx. Additionally, NLRP3 activation induced by nigericin, alum, monosodium urate crystals, and the membrane-attack complex have been shown to depend on Ca^2+^ flux and K^+^ efflux (Triantafilou et al., 2013). Mitochondrial dysfunction, leading to the release of mtROS and mitochondria DNA (mtDNA) into the cytosol, is an additional key upstream event implicated in NLRP3 activation (Zhong et al., 2018). Differently, RNA viruses activate NLRP3 through mitochondrial antiviral signaling proteins (MAVs) on the mitochondrial outer membrane and viral proteins are recognized under the help of DNA-dependent activator of IRFs/Z-DNA binding protein 1(DAI/ZBP1; Kuriakose et al., 2016). Disruption of the lysosoma membrane caused by phagocytosis of particulate matter or live pathogens is also linked with NLRP3 activation (Dostert et al., 2008). Though many pathways are interrelated and overlapped, all signals induce NIMA-related kinase 7 (NEK7) to bind with NLRP3, and then initiate inflammasome assembly (Sharif et al., 2019). Assembly of the NLRP3 inflammasome leads to autoproteolytic cleavage of pro-caspase 1, and the activated caspase 1 mediates the release of the inflammatory cytokines IL-1β and IL-18, as well as GSDMD-mediated pyroptotic cell death, which is a rapid, inflammatory form of lytic programmed cells (He et al., 2015).

**FIGURE 1:**
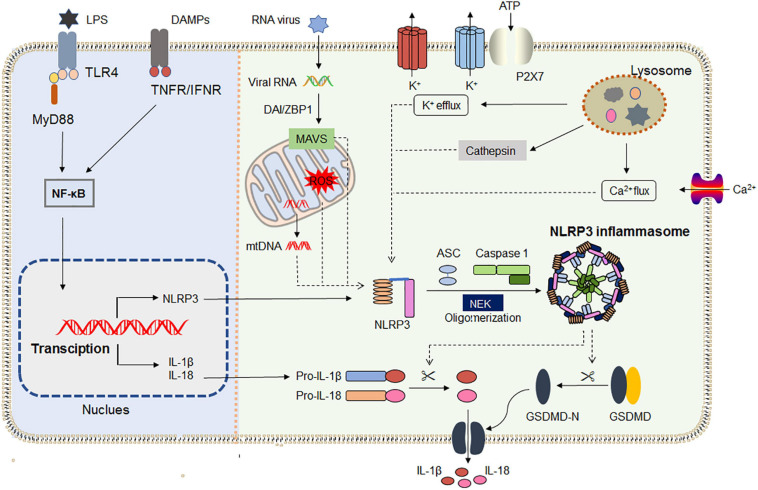
NLRP3 inflammasome priming and activation. The priming **(left)** is provided by activation of pathogen-associated molecular patterns (PAMPs) or cytokines, leading to the transcriptional upregulation of NLRP3, IL-1, and IL-18 in a NF-κB-dependent way. Those genes’ transcriptions are induced through the recognition of engage pattern-recognition receptors. Activation **(right)** is provided by any numerous PAMPs or damage-associated molecular patterns (DAMPs), such as particulates, ATP, crystals, and pathogens, which activate multiple upstream signaling events including K^+^ efflux, Ca^2+^ flux, Cl^–^ efflux, lysosomal disruption, ROS production, and mtDNA. All signals induce NIMA-related kinase 7 (NEK7) to bind with NLRP3, and then initiate inflammasome assembly. Assembly of the NLRP3 inflammasome leads to autoproteolytic cleavage of pro-caspase 1, and the activated caspase 1 mediates the release of the inflammatory cytokines IL-1β and IL-18, as well as GSDMD-mediated pyroptosis. IFNAR, IFNα/β receptor; IL-1R1, IL-1 receptor type 1; NF- κB, nuclear factor- κB; NEK7, NIMA- related kinase 7; MAVS, antiviral signaling protein; DAI/ZBP1, DNA-dependent activator of IRFs/Z-DNA binding protein 1.

Recent research shows that NLRP3 inflammasome activation does not always follow the classical two-step activation model. The lipopolysaccharide (LPS) of Gram-negative bacteria could be directly recognized by the CARD domain of caspase-4/5/11, resulting in its oligomerization, and then the active caspase-4/5/11 cleaves the pore-forming protein GSDMD (Xing et al., 2017; Hu et al., 2019). Moreover, NLRP3 inflammasome assembles upon TLR4 activation by LPS triggering the TIR-domain-containing adapter-inducing interferon/receptor-interacting serine/threonine-protein kinase 1/Fas-associated protein with death domain/caspase-8 signaling cascade, which leads to the gradual release of IL-1β but does not induce pyroptosis (Gaidt et al., 2016). The latest research found that dynein adapter histone deacetylase 6 was indispensable for the microtubule transport and assembly of NLRP3 inflammasome *in vitro* and *in vivo* (Magupalli et al., 2020). Samir et al. (2019) verified that DEAD-box helicase 3X (a known component of stress granules) acted as a “live-or-die” checkpoint in stressed cells by regulating NLRP3 inflammasome. NLRP3 inflammasome biology has been widely exploited in the past two decades. The general mechanisms by which NLRP3 inflammasome is primed and activated are summarized in Figure 1.

## Drug-Induced Toxicity in Different Organs

**FIGURE 2:**
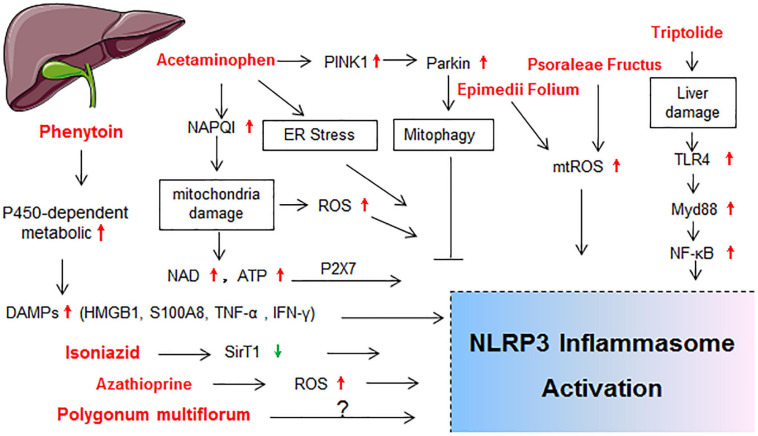
Schematic diagram showing the molecular mechanisms of NLRP3 activation in drug-induced liver injury. BMDMs, bone marrow derived macrophages; ER stress, endoplasmic reticulum stress; HMGB1, high mobility group protein; IL-1R1, IL-1 receptor type 1; LSECs, liver sinusoidal endothelial cells; NAD; nicotinamide adenine dinucleotide; NLRP3 inflammasome, including NLRP3, ASC and caspase-1; TLR4, toll-like receptor 4; TNF-α, tumor necrosis factor-alpha; TXNIP; thioredoxin-interacting protein.

**FIGURE 3:**
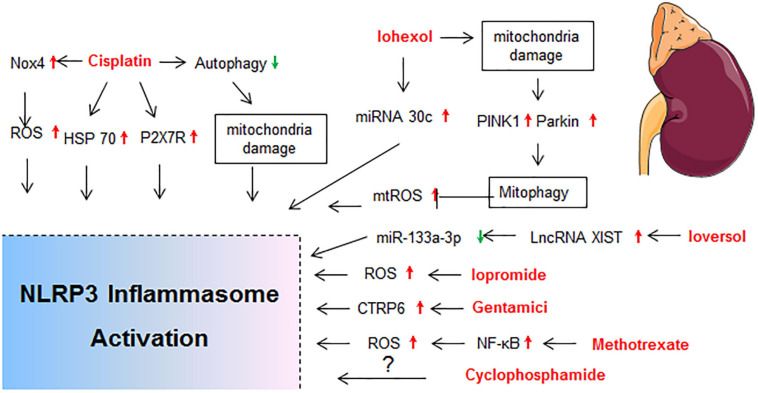
Molecular mechanisms of NLRP3 activation in drug-induced nephrotoxicity. BNIP3, BCL2/adenovirus E1B interacting protein 3; HIF-1α, hypoxia inducible factor 1, alpha subunit; HSP70, heat shock protein 70; LncRNA XIST, long non-coding RNA X-inactive specific transcript; MTECs, mouse tubular epithelial cells; P2X7R, P2X purinoreceptor 7 receptor; TECs, tubular epithelial cells.

**FIGURE 4:**
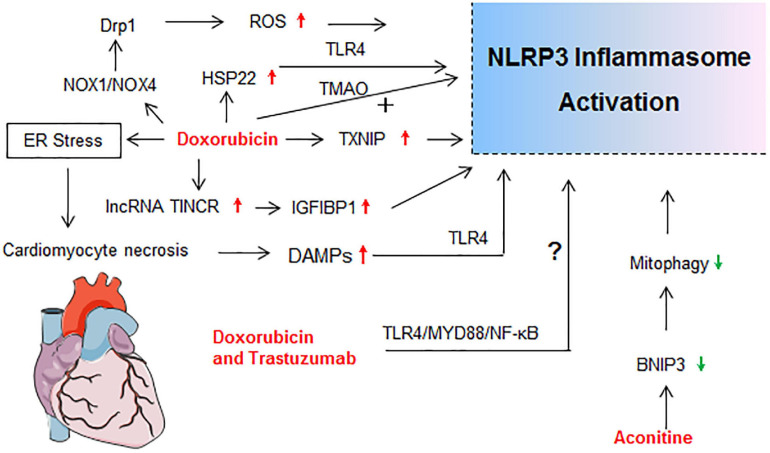
Molecular mechanisms of NLRP3 activation in drug-induced cardiotoxicity. drp1, dynamin-related protein 1; ER stress, endoplasmic reticulum stress; HSP22, heat shock protein 22; IGF2BP1, insulin like growth factor 2 mRNA binding protein 1; lncRNA TINCR, terminal differentiation-induced non-coding RNA; NOX, nicotinamide adenine dinucleotide phosphate oxidase; TMAO, Trimethylamine N-oxide.

**FIGURE 5:**
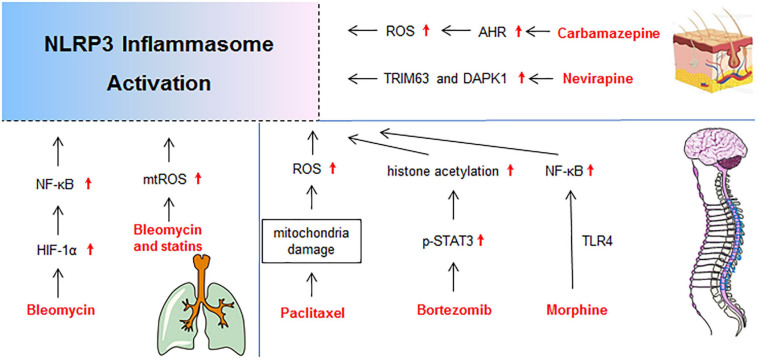
Molecular mechanisms of NLRP3 activation in drug-induced gutaneous reaction, neurotoxicity, and lung injury. DAPK1, death-associated protein kinase 1; p-STAT3, phosphorylation-signal transducer and activator of transcription-3; TRIM63, Tripartite Motif-containing Protein 63.

Drug-induced toxicity appears in multiple organs, but the liver and kidney are the most common targets due to their vital roles in the metabolism and excretion of drugs. The heart, lung, skin, gastrointestinal tract, hematological system, and nervous system are also probable targets for drug-induced toxicity. In the following sections, detailed mechanisms that related to NLRP3 inflammasome activation in diverse organ injury induced by drugs listed in Supplementary Table 1 are discussed. Molecular mechanisms of NLRP3 activation in drug-induced liver injury, nephrotoxicity, cardiotoxicity, and others are described independently in Figures 2–5, respectively.

### Drug-Induced Liver Injury

Liver is the major organ for metabolism and many drugs are metabolized through the liver cytochrome p450 enzymes (CYP450s; Ingelman-Sundberg, 2004). Hence, drug-induced liver injury (DILI) represents one main reason for the cessation of new drug development and withdrawal of an approved drug from the market (Pugh et al., 2009; Yano et al., 2014). The activation of NLRP3 inflammasome in hepatocytes and non-parenchymal cells resulted in hepatocyte pyroptosis, induction of proinflammatory signaling, and liver fibrosis, which finally exacerbated the progression of various liver pathologies (Wree et al., 2014; Gaul et al., 2021). The molecular mechanisms of NLRP3 activation in DILI are summarized in Figure 2.

#### Acetaminophen-Induced Liver Injury

Acetaminophen (N-acetyl-*p*-aminophenol, also known as paracetamol) is a widely used safe antipyretic and analgesic drug. However, acetaminophen overdose is the most frequent cause of acute liver failure in the United States and many European countries (Larson et al., 2005; Yano et al., 2014). Severe liver injury, along with increasing expressions of NLRP3 and IL-1β, occurred in acetaminophen-treated mice liver or liver cell lines (Chen H. et al., 2020; Lv et al., 2020; Samra et al., 2020; Shi et al., 2020). Imaeda et al. (2009) found that genetic deficiencies in NLRP3 inflammasome components (NLRP3, ASC, and caspase-1) provided protection from acetaminophen-induced mice death and liver injury. Both ATP and nicotinamide adenine dinucleotide (NAD) have been reported to be up-stream regulators of the NLRP3 inflammasome (Liang et al., 2019). P2X7R activation provoked by ATP and NAD was required for acetaminophen-induced hepatotoxicity in mice (Sohail et al., 2010; Hoque et al., 2012). Compared with wild-type (WT) mice, P2X7^–/–^mice showed significantly decreased liver necrosis. Pretreatment with specific P2X7 antagonist or competitive NAD antagonist significantly decreased acetaminophen-induced necrosis and hemorrhage in liver injury as well (Hoque et al., 2012). Acetaminophen overdose also resulted in the activation of PTEN-induced putative kinase 1(PINK1)/Parkin-mediated mitophagy in mouse liver (Shan et al., 2019). Pretreatment with autophagy inductor (rapamycin) alleviated acetaminophen-induced hepatoxicity and significantly inhibited the activation of NF-κB, NLRP3 inflammasome, and the production of IL-1β (Shan et al., 2019). In another study, acetaminophen promoted autophagy by modulating endoplasmic-reticulum-stress-mediated NLRP3 inflammasome activation (Kim and Park, 2018). As autophagy can remove the damaged and dysfunctional mitochondria and reduce mtROS generation, it implies that autophagy may act as a negative regulator of NLRP3 activation (Qu et al., 2019b; Shan et al., 2019). Consistent with those observations, several studies found that IL-1β mRNA and protein levels were increased in the liver and serum of acetaminophen treated mice (Blazka et al., 1995; Dambach et al., 2002; Jaeschke et al., 2006; Ishibe et al., 2009; Williams et al., 2010). Administration of anti-IL-1β antibody also improved survival of mice after a lethal dose of acetaminophen administration (Imaeda et al., 2009).

Though evidence has shed important light on the role of NLRP3 inflammasome in acetaminophen hepatotoxicity, there are also some controversial conclusions. A small-sample-sized study of children and adolescents with acetaminophen hepatotoxicity found that acetaminophen overdose was associated with increased serum IL-8 rather than IL-1β (James et al., 2001). In addition, the overall formation of IL-1β after acetaminophen overdose was limited in both humans (Woolbright et al., 2015) and mice (Imaeda et al., 2009), and the increase was disproportionally small compared with the death of hepatocyte (Williams et al., 2010). It has been reported that NLRP3 inflammasome components gene knock-out mice exhibited reduced hepatoxicity after acetaminophen treatment (Imaeda et al., 2009), which could not be reproduced under a lower dose of acetaminophen (300 mg/kg versus 500 mg/kg) (Williams et al., 2011). But the interleukin 1-receptor 1(IL-1R1) deficient mice did not show reduced inflammation and injury induced by acetaminophen (Williams et al., 2010). Moreover, a substantial dose of IL-1β (20 μg/kg) administered after acetaminophen led to the recruitment of neutrophils into the liver but did not enhance acetaminophen-induced hepatoxicity (Williams et al., 2010). Several studies aiming at inhibiting the activation of caspase-1 by using pan-caspase inhibitor also did not show protection (Lawson et al., 1999; Clemens, 2006; Williams et al., 2010). It is widely accepted that the hepatotoxicity of acetaminophen depends on the reactive metabolite N-acetyl-p-benzoquinone imine (NAPQI) within hepatocytes and sinusoidal endothelial cells. NAPQI initiates a cascade of intracellular events resulting in the damage or even death of hepatocytes (Nelson, 1990; Holt et al., 2010). However, whether NAPQI is involved in the activation of NLRP3 inflammasome in acetaminophen-induced liver injury has not yet been elucidated. The role of NLRP3 inflammasome in acetaminophen hepatotoxicity is complicated and further verification and evaluation are needed.

#### Azathioprine-Induced Liver Injury

A prospective cohort study reported that hepatotoxicity of azathioprine, an immunosuppressive drug, was recognized in about 2% of patients with psoriatic arthritis and rheumatoid arthritis within weeks after the initiation of azathioprine treatment (Takatsu et al., 2009; Aithal, 2011). The mRNA expressions of NLRP3, IL-1β, TNF-α, and macrophage inflammatory protein 2 in the liver were significantly increased in mice that were orally administered azathioprine at 300 mg/kg, which was 40-fold higher than the clinical dose (Matsuo et al., 2014). Moreover, a small aggregation of neutrophils in the hepatic parenchyma was found, indicating that NLRP3 inflammasome activation induced by azathioprine contributed to the innate immune reaction and liver injury (Matsuo et al., 2014).

#### Busulfan and Cyclosphamide-Induced Liver Injury

It has been reported that busulfan combined with cyclosphamide (BU/CY) used before hematopoietic stem cell transplantation (HSCT) may cause damage to liver sinusoidal endothelial cells and liver dysfunction (Qiao et al., 2015a). Increased expressions of NLRP3, caspase-1, IL-1β, and IL-18 were observed after transplantation conditioned with BU/CY in mice, which was consistent with the hepatic pathological changes. BAY 11-7082 (a NLRP3 inhibitor) pretreatment ameliorated liver inflammation and improved the liver function (Qiao et al., 2015b). Importantly, ATP level was positively correlated with NLRP3 expression, suggesting that ATP might be the potential priming signal to NLRP3 inflammasome activation in the liver after HSCT (Qiao et al., 2015b).

#### Carbamazepine-Induced Liver Injury

Carbamazepine is a widely used antiepileptic agent and it could cause rare but severe idiosyncratic liver injury in humans (Bjornsson and Olsson, 2005; Chalasani et al., 2015). In the liver, carbamazepine is majorly oxidized to 2-hydroxycarbamazepine and 10, 11-epoxide by CYP450s. *In vitro*, THP-1 cells (a human macrophage cell line) incubated with 2-hydroxyiminostilbene or the supernatant from hepatocytes with carbamazepine led to an increase of caspase-1 activity and release of IL-1β (Kato et al., 2019). While carbamazepine or LPS alone has no effects on liver structure and function in WT and NLRP3^–/–^ mice, combination of carbamazepine and LPS led to liver injury in WT mice but not in NLRP3^–/–^mice, indicating that carbamazepine could induce hepatotoxicity via promoting NLRP3 inflammasome activation *in vivo* (Wang Z. et al., 2019). Additionally, carbamazepine enhanced NLRP3-mediated caspase-1 activation and IL-1β maturation in cultured bone marrow derived macrophages (BMDMs) by synergistic induction of mtROS production. Hepatic parenchymal cells pyroptosis, as well as monocytes/macrophages or neutrophils infiltration induced by carbamazepine, all contributed to the progress of hepatotoxicity (Wang Z. et al., 2019).

#### *Epimedii Folium* and *Psoraleae Fructus*-Induced Liver Injury

*Epimedii Folium* combined with *Psoraleae Fructus* is a common traditional Chinese medicine preparation used to invigorate the liver and kidney. Both *Epimedii Folium* and *Psoraleae Fructus* could induce liver injury according to clinical research (Wang et al., 2016; Melchart et al., 2017). Combination of non-hepatotoxic doses of LPS and icariside II (the major active and metabolic constituent of *Epimedii Folium*) causes the increase of aminotransferase activity, hepatic inflammation, and pyroptosis, which is attenuated by Nlrp3 deficiency or pretreatment with MCC950 (a specific NLRP3 inhibitor) *in vivo* and *in vitro* (Wang et al., 2020). In another study (Wang et al., 2021), eight bioactive compounds involved in *Psoraleae Fructus*-induced NLRP3 inflammasome activation, among which only psoralidin activated the NLRP3 inflammasomes, followed by secreting caspase-1 and IL-1β in a dose-dependent manner in normal mice. Psoralidin further induced hepatic inflammation, increased aminotransferase activity, and increased the production of IL-1β and TNF-α in a LPS-mediated susceptible mouse model (Wang et al., 2021). Interestingly, psoralidin significantly induced IL-1β maturation and caspase-1 activation in NLRP3-knockout BMDMs, indicating that psoralidin also activated other types of inflammasomes (Wang et al., 2021). Similarly, either *Epimedii Folium* or *Psoraleae Fructus* alone or in combination led to liver injury, while *Epimedii Folium* or *Psoraleae Fructus* alone could lead to liver injury in LPS-treated rats (Gao et al., 2020). Furthermore, *Epimedii Folium* or *Psoraleae Fructus* alone or in combination enhanced the LPS-stimulated IL-1β production, which is processed and released by activating the NLRP3 inflammasome (Gao et al., 2020). Therefore, activation of NLRP3 inflammasome aggravated the idiosyncratic hepatotoxicity induced by *Epimedii Folium* or *Psoraleae Fructus* under immunological stress conditions.

#### Isoniazid-Induced Liver Injury

Clinical application of the first-line anti-tuberculosis drug isoniazid is also limited due to severe hepatotoxicity (Russom et al., 2018). Apart from the liver histopathological deterioration, isoniazid induced NLRP3 inflammasome activation in a sirtuin 1 (SIRT1)-dependent manner, led to liver injury in rats and L02 cells (Zhang Y. M. et al., 2020). Isoniazid also increased cell apoptosis by elevating the expressions of p53, Bax, and cleaved-caspase 3 (Zhang Y. M. et al., 2020). However, whether activation of NLRP3 inflammasome mediates the cell apoptosis remains unclear yet.

#### Phenytoin Isoniazid-Induced Liver Injury

Phenytoin is an anticonvulsant drug that is widely used in the treatment of epilepsy. The patients who are administered phenytoin may suffer drug hypersensitivity, such as rash, fever, and even drug-induced liver injury characterized by hepatic necrosis (Mullick and Ishak, 1980; Velasco and McDermott, 2014). The levels of plasma IL-1β and high mobility group protein (HMGB1, a late proinflammatory factor) elevated with the increase of liver NLRP3 and IL-1β mRNA expressions in the mice model. NLRP3 inflammasome activation leading to IL-1β and HMGB1 production contributed to the onset of liver inflammation and injury (Sasaki et al., 2013).

#### *Polygonum multiflorum*-Induced Liver Injury

*Polygonum multiflorum*, the root of *P. multiflorum* Thunb, has been used for several decades as an herbal treatment for constipation and early graying of the hair. *P. multiflorum* was initially considered to be non-toxic, but clinical reports of hepatotoxicity have increased (Dong et al., 2014). According to the screening and analysis of different extraction sites of *P. multiflorum*, the hepatotoxicity-related components were determined by 2,3,5,4′-tetrahydroxy-cis-stilbene-2-O-β-glucoside (Cis-SG) and 2,3,5,4′-tetrahydroxy-trans-stilbene-2-O-β-glucoside (Trans-SG; Li et al., 2017). Cis-SG induces immunological idiosyncratic hepatotoxicity in LPS-treated rats by suppressing PPAR-γ (Meng et al., 2017). Though Trans-SG could not induce immune stress-mediated liver injury directly, it enhanced liver sensitivity to Cis-SG and led to liver injury (Li et al., 2017). In a research combination of 3 D cultured HepG2 cells and THP-1 macrophages, secretion of IL-1β and the expressions of ASC, NLRP3, caspase-1, and IL-1β significantly were increased in THP-1 derived macrophages incubated by supernatants from HepG2 cells incubated with Cis-SG or Trans-SG (Pan et al., 2021). The combination of 3 D cultured HepG2 cells and THP-1 macrophages is a convenient way to test the ability of drugs to activate NLRP3 inflammasome, which may contribute to screening drugs with liver injury risks and provide a method for the prediction and solution of drug-induced hepatotoxicity (Pan et al., 2021).

#### Triptolide-Induced Liver Injury

Triptolide is the major active compound derived from the traditional Chinese medicine *Tripterygium wilfordii Hook*, which exhibited excellent immunomodulatory and anti-tumor activities. But the application of triptolide was restricted due to its narrow therapeutic window and hepatotoxicity (Ziaei and Halaby, 2016). Mice treated with a single dose of triptolide (600 μg/kg) displayed liver injury with a time-dependent activation of NLRP3 inflammasome, accompanied by the increase of serum transaminases and elevation of neutrophils infiltration. The activation of TLR4-Myd88-NF-κB pathway and oxidative stress induced by triptolide might be responsible for the activation of NLRP3 inflammasome (Yuan et al., 2019). And caspase-1 inhibitor (Ac-Yvad-Cmk) pretreatment effectively decreased the recruitment of neutrophils and inhibited the production of massive pro-inflammatory factors (IL-1β, TNF-α, IL-6, and MCP1; Yuan et al., 2019). Those findings suggested that triptolide induced hepatotoxicity mainly through the proinflammatory effects mediated by NLRP3 inflammasome.

Considering the low incidence and unpredictability of idiosyncratic drugs’ hepatotoxicity, Yano et al. (2014) established a novel cell-based screening system to analyze the comprehensive gene expressions of four sets of hepatotoxic and non-hepatotoxic drugs during DILI in mice. The hepatic mRNA levels of S100A8, S100A9, NALP3, IL-1β, and the receptor for advanced glycation end products (RAGE) were commonly increased in hepatotoxic drug-administered mice compared with those in non-hepatotoxic drug administered mice. In HL-60 or K562 cells incubated with human liver microsomes, the total sum score of expression levels of five genes mentioned above *in vivo* could help identify clinical drugs at high risk for hepatotoxicity (Yano et al., 2014). NLRP3 molecule can be used as a candidate biomarker for the risk assessment of DILI in the preclinical development and clinical application of drugs.

### Nephrotoxicity

The kidney is a frequent site of drug toxicity due to its role in the excretion of drugs and toxic agents. Nephrotoxin administration is the second cause of hospital acquired acute kidney injury (AKI; Nash et al., 2002), and accounts for 19–26% of adult cases and 16% of pediatric cases of AKI (Chawla et al., 2014; Mehta et al., 2015). Many kidney diseases exhibited increased expression of NLRP3 mRNA in kidney tissue from human renal biopsies, and the NLRP3 mRNA level was correlated with renal function (Vilaysane et al., 2010). The molecular mechanisms of NLRP3 activation in drug-induced nephrotoxicity is summarized in Figure 3.

#### Aristolochic Acid-Induced Nephrotoxicity

Aristolochic acid-induced nephropathy (AAN) is a tubulointerstitial disease caused by ingestion of Chinese medicinal herbs containing aristolochic acid (Jadot et al., 2017). Recent investigations showed that NLRP3 inflammasome was activated in both mouse kidney and cultured HK-2 cells after aristolochic acid exposure. NLRP3 or caspase-1 deficiency protected mice from renal injury in an acute AAN model (Wang S. et al., 2019). Aristolochic acid treatment also promoted the expression of NLRP3 and α-SMA, increased the secretion of caspase-1, IL-1β, and IL-18, and inhibited the expression of E-cadherin in HK-2 cells in a dose- and time-dependent manner, leading to the trans-differentiation of renal tubular epithelial cells (Yu M. et al., 2020).

#### Cisplatin-Induced Nephrotoxicity

Cisplatin is the most widely studied drug with high hepatotoxicity, and 20% of patients receiving cisplatin show nephrotoxicity (Manohar and Leung, 2018). Increased NLRP3 protein expression was observed in both renal tubular and glomerulus cells upon exposure to cisplatin in rats (Qu et al., 2019b). Activation of NLRP3 inflammasome was also found in mouse models of cisplatin-induced AKI (Ullah et al., 2020; Yang et al., 2020), which was positively regulated by heat shock protein 70 (Ullah et al., 2020) or mtROS (Ma et al., 2019; Yang et al., 2020). The expressions of P2X7R, NLRP3 inflammasome components, IL-1β, and IL-18 were all significantly upregulated in the renal tubular epithelial cells of C57BL/6 mice after cisplatin treatment (Zhang et al., 2014). Pretreatment with P2X7R antagonist (A-438079) significantly alleviated cisplatin-induced renal histological damage, inflammatory response, and apoptosis and improved renal function, which were associated with the levels of NLRP3, ASC, caspase-1, oxidative stress, p53, and caspase-3 (Zhang et al., 2014). In a rat model reflecting AKI progression to renal interstitial fibrosis induced by cisplatin, the levels of ROS, α-SMA, NLRP3, and cleaved caspase-3 in renal tissue were significantly increased (Li et al., 2020). In addition, autophagy reduced the assembly of NLRP3 inflammasome in cisplatin-induced liver and kidney acute injury (Qu et al., 2019a).

Renal tubular epithelial cell is thought to be the major target of cisplatin for renal toxicity, and the exposure to cisplatin activates complex signaling pathways that lead to injury and death of tubular cells as well as a robust inflammatory response, which further exacerbates renal tissue damage (Schrier, 2002; Fukasawa et al., 2014). Several studies supported that the NLRP3 inflammasome activation in tubular cells contributed to the development of cisplatin nephrotoxicity. Charles L. Edelstein demonstrated that caspase-1 was a mediator of cisplatin-induced AKI; caspase-1 deficient mice were protected from cisplatin-induced apoptosis and acute tubular necrosis (Faubel et al., 2004). Pan-caspase inhibitor (QVD-OPH) co-treatment also protected cells from necrosis in cisplatin-treated freshly isolated proximal tubules, and the protection was associated with a decrease in caspase-1, IL-1α, and IL-1β (Lee et al., 2015). However, opposite views and results were reported. The NLRP3, caspase-1, IL-1α, and IL-1β were activated in proximal tubules of cisplatin-treated mice, but expression of NLRP3 was not increased by cisplatin (Lee et al., 2015). Interestingly, NLRP3^–/–^ mice were protected from ischemic but not cisplatin-induced AKI (Kim et al., 2013). Immunoblot analysis of whole kidney after cisplatin-induced AKI revealed an increase in ASC, caspase-1 activity, caspase-5, NLRP1, and NLRP3. Besides, increased caspase-1, IL-1β, and IL-1α were also observed in proximal tubules of mice treated with cisplatin. However, the increase of caspase-1 in kidney and proximal tubules was not associated with a statistical increase in NLRP3 protein (Kim et al., 2013). The role of other inflammasomes, such as NLRP1 inflammasome, demands further study into cisplatin-induced AKI (Kim et al., 2013).

#### Contrast-Induced AKI

Contrast-induced AKI occurred in more than 30% of patients receiving iodinated contrast media injection, and it is associated with a high risk of mortality due to renal failure (Fähling et al., 2017). The mechanism of contrast-induced AKI mainly includes vasoconstrictor release, oxidative stress, and direct cytotoxic effects on renal tubular or vascular endothelial cells (Scharnweber et al., 2017). Expressions of NLRP3 and ASC were significantly upregulated with the treatment of contrast media (isopaque or omnipaque) in HK-2 cells and unilateral nephrectomy model. Silence of NLRP3 or ASC attenuated contrast-induced apoptosis, as well as the secretion of IL-1β and IL-18 (Shen et al., 2016). The RNA sequencing analysis of renal cortex revealed that the *nlrp3* or *caspase-1* knockout iohexol-induced AKI mice exhibited upregulated cellular response to hypoxia, mitochondrial oxidation, and autophagy when compared with WT iohexol-induced AKI mice, which indicated that NLRP3 inflammasome inhibition resulted in the upregulation of hypoxia signaling pathway and mitophagy (Lin Q. et al., 2020). BNIP3 (BCL2/adenovirus E1B interacting protein 3) is a pro-apoptotic protein and the cooperation of BNIP3 and LC3-II can mediate autophagy to remove damaged mitochondria under certain conditions (Fu et al., 2020). The *nlrp3* or *caspase-1* knockout iohexol-induced AKI mice and iohexol-treated HK-2 cells with MCC950 pretreatment exhibited upregulated levels of HIF-1α (hypoxia inducible factor 1, alpha subunit), BNIP3, and LC3B-II, as well as enhanced colocalization of LC3B with BNIP3 and mitochondria (Lin Q. et al., 2020). NLRP3 inflammasome activation promoted apoptosis and downregulated HIF1α and BNIP3-mediated mitophagy in iohexol-induced AKI, which eventually aggravated renal injury (Lin Q. et al., 2020). Similarly, iohexol caused mitochondrial damage of renal tubular epithelial cells and induced mtROS production and NLRP3 inflammasome activation, which were reduced by PINK1/Parkin-mediated mitophagy (Lin et al., 2019). Activation of S100A8/A9/TLR4/NLRP3 inflammasome axis was also found to contribute to iopromide-induced inflammation, apoptosis, and ROS generation in rat kidneys (Tan et al., 2017).

It has been demonstrated that microRNA-30 was upregulated in the contrast-induced AKI (Gutierrez-Escolano et al., 2015) and NLRP3 inflammasome was targeted by microRNA-30. In cultured pig renal epithelial cells, iohexol promoted cell apoptosis, NLRP3 expression, caspase-1 cleavage, and IL-1β secretion, which were reversed by microRNA-30 mimic. MicroRNA-30 may act as a negative feedback regulator and suppress the renal injury and fibrosis via targeting NLRP3 (Xu et al., 2020). In many diseases, long non-coding RNA**s** (lncRNAs) serve as miRNA “sponges,” and miRNAs could regulate lncRNAs and compete for mRNAs (Chen et al., 2018). Bioinformatic analysis found that miR-133a-3p could bind with X-inactive specific transcript (XIST) and target NLRP3 at the complementary binding sites (Liu et al., 2021). In the rat contrast-induced nephropathy model, ioversol induced kidney morphology changes, with increases on SCr and BUN contents, elevated levels of IL-1β and IL-18, upregulated expressions of lncRNA XIST, NLRP3, ASC, and cleaved caspase-1, and decreased miR-133a-3p mRNA expression (Liu et al., 2021). Moreover, downregulating XIST attenuated ioversol-induced AKI via regulating miR-133a-3p/NLRP3 axis. XIST and NLRP3 can be used as a potential marker for contrast-induced AKI amelioration (Liu et al., 2021).

In addition, caspase-4/5/11 was required for iohexol-induced IL-1β cleavage and pyroptosis in human and mouse tubular epithelial cells (Shi et al., 2015; Zhang Z. et al., 2018). And renal tubular epithelial caspase-11 activation was required for generation of cleaved GSDMD. However, which inflammasome is involved in the tubular epithelial cells pyroptosis remains unclear (Zhang Z. et al., 2018). Lau et al. (2018) reported that the cleaved caspase-1 was up-regulated in the human urine pellets after contrast administration. Levels of IL-18 and the kidney injury marker-1 in urine were increased immediately after ioversol administration in patients undergoing coronary angiography. NLRP3-deficient mice displayed reduced kidney epithelial cell injury and inflammation in ioversol-induced AKI, however, ioversol-induced tubular epithelial cell death was not dependent on the NLRP3 *in vitro* (Lau et al., 2018). Further contrast uptake data revealed that ioversol activated the canonical NLRP3 inflammasome in the macrophages and ioversol-induced AKI was dependent on resident renal phagocytes, IL-1β, leukocyte recruitment, and dipeptidase-1 (Lau et al., 2018). Therefore, contrast-induced AKI is a multistep process that involves damage of tubular epithelial cells, renal resident cells, and infiltrating cells. Both NLRP3 inflammasome-dependent inflammation and pyroptosis induced by other inflammasomes contributed to the progress of nephrotoxicity.

#### Gentamicin-Induced AKI

At least 10–25% of patients receiving therapeutic doses of gentamicin (an aminoglycoside) are at an increased risk of developing AKI. Previous studies supported that gentamicin-induced renal damage was linked with a significant increase of oxidative stress of renal cortex (Balakumar et al., 2010). Expressions of NLRP3 and caspase-1 in kidney, along with the secretion of IL-1β and TNF-α, were increased in rats after gentamicin treatment. Acting as an anti-inflammatory factor, C1q/tumor necrosis factor related protein 6 (CTRP6) reversed the activation of NLRP3 inflammasome in a dose dependent manner (Li et al., 2016).

#### Methotrexate-Induced AKI

The dihydrofolate reductase inhibitor methotrexate and its 7-hydroxy metabolite tend to crystallize and precipitate in the renal tubules, which leads to nephrotoxicity (Perazella, 2009). Several studies have demonstrated AKI in 2–12% of patients who received high dose methotrexate (May et al., 2014; Howard et al., 2016). In Wistar rats, methotrexate induced the overproduction of ROS and upregulated the NF-κB p65, which activated NLRP3 inflammasome and its down-stream proteins caspase-1 p20 and IL-1β in the kidney (Abd El-Twab et al., 2019; Mahmoud et al., 2019).

### Cardiotoxicity

Cardiotoxicity ranks among the most serious adverse side effects of clinically used drugs. Cardiotoxicity, including acute and late-onset cardiotoxicity, is a well-known adverse effect of many types of antitumor agents (especially the anthracycline) and some traditional Chinese medicines (Qiu et al., 2019; Cardinale et al., 2020). The molecular mechanisms of NLRP3 activation in drug-induced cardiotoxicity is summarized in Figure 4.

#### Aconitine-Induced Cardiotoxicity

Aconitine, a natural product extracted from Aconitum species, is widely used for treating rheumatism, arthritis, bruises, and fractures. However, cardiotoxicity and neurotoxicity caused by aconitine have been reported (Lin et al., 2004). Recent exploration demonstrated that the aconitine cytotoxicity in cardiomyocytes depended on the activation of the TNF-α and NLRP3 inflammasome pathways (Peng et al., 2020). The BNIP3-dependent mitophagy potently alleviated myocardial injuries of aconitine *in vitro* and *in vivo* by mitigating the activation of TNF-α/NLRP3 inflammasome signaling axis. Elevating the BNIP3-dependent mitophagy and inhibiting the TNF-α/NLRP3 inflammasome signaling pathway may provide novel insights into the prevention of aconitine-related toxicity (Peng et al., 2020).

#### Doxorubicin-Induced Cardiotoxicity

It is well known that administration of anthracyclines, especially doxorubicin, is associated with acute and late cardiotoxicity, leading to increased risk of heart failure (van der Zanden et al., 2020). Kobayashi et al. (2016) reported that NLRP3 deficiency enhanced the susceptibility to doxorubicin-induced cardiotoxicity independent of IL-1β, which was proven by the results that cardiac dysfunction and injury were induced by doxorubicin (15 mg/kg) administration in NLRP3^–/–^mice but not in WT and IL-1β^–/–^mice. However, this view is fuzzy and questionable. Their subsequent data showed that NLRP3 regulated IL-10 production in macrophages, which contributed to the progress of doxorubicin-induced cardiotoxicity (Kobayashi et al., 2016). Many investigations have proven that doxorubicin at 15 mg/kg was sufficient to establish a cardiotoxicity mouse model. Pharmacological inhibition of the NLRP3 inflammasome also limited the left ventricle systolic dysfunction and myocardial cells’ death after doxorubicin exposure in mice or rats (Marchetti et al., 2015; Sun et al., 2020). Recent research highlighted the important role of NLRP3 inflammasome in doxorubicin-induced cardiotoxicity (Figure 4).

Doxorubicin treatment significantly increased cardiac expression of TLR4, NLRP3, caspase-1, IL-1β, IL-18, TNF-α, and cell signaling proteins (MyD88, p-P38, and p-JNK) in mice (Singla et al., 2019). It has been reported that terminal differentiation-induced non-coding RNA (lncRNA TINCR) could attenuate cardiac hypertrophy by epigenetically silencing CaMKII and insulin like growth factor 2 mRNA binding protein 1 (IGF2BP1) could participate in the modification of mRNA stability (Shao et al., 2017; Meng et al., 2019). Meng et al. (2019) first demonstrated that lncRNA TINCR acted as a critical upstream regulatory factor in doxorubicin-induced cardiomyocyte NLRP3 inflammasome activation and pyroptosis in a TINCR/IGF2BP1/NLRP3-dependent manner. Doxorubicin increased TINCR expression by inducing H3K27 acetylation at the promoter region of TINCR gene and activating transcription in cardiomyocytes. Enhanced TINCR upregulated NLRP3 expression through increasing mRNA stability via IGF2BP1, resulting in the activation of caspase-1 and GSDMD pathways (Meng et al., 2019). In dilated cardiomyopathy model induced by doxorubicin, NLRP3 inflammasome activation and pyroptosis occurred in doxorubicin-treated heart tissue, but were of very low levels in either NLRP3^–/–^ or caspase-1^–/–^ mice (Zeng et al., 2020). Doxorubicin enhanced expressions of NOX1 [nicotinamide adenine dinucleotide phosphate (NADPH) oxidase 1] and NOX4 and induced mitochondrial fission through dynamin-related protein 1 activation, leading to NLRP3 inflammasome-mediated pyroptosis in cardiomyocytes via a caspase-1-dependent manner (Zeng et al., 2020). Our study showed that doxorubicin induced mice myocardium and cardiomyocytes apoptosis concomitantly with ROS over-production, and up-regulation of NLRP3, ASC, and caspase-1 p20 expressions, as well as increased IL-1β and LDH secretion in cardiomyocytes (Wei et al., 2020). In addition, doxorubicin-induced H9c2 cardiomyocyte senescence was dependent on thioredoxin-interactive protein (TXNIP)/NLRP3 inflammasome/p16^INK4A^/p21 signaling pathway (Huang et al., 2020).

Trimethylamine N-oxide (TMAO) is a gut microbiota-dependent metabolite of specific dietary nutrients, which is a cardiovascular risk biomarker linked to cardiac fibrosis, coronary atherosclerosis, and heart failure (Berger et al., 2020). Moreover, TMAO could promote vascular inflammation through mitogen-activated protein kinase and NF-κB signaling (Seldin et al., 2016). TMAO or doxorubicin alone could activate NLRP3 inflammasome *in vivo* (Li et al., 2019). Further, TMAO exacerbated doxorubicin-induced cardiac dysfunction and cardiac fibrosis by elevating collagen accumulation, profibrotic levels, and inflammatory factors via promoting NLRP3 inflammasome activation (Li et al., 2019). Apart from increasing NLRP3 inflammasome, the level of Heat shock protein 22 (Hsp22), which exerted anti-apoptosis and anti-inflammatory effects, was found to be increased in doxorubicin-treated heart tissue. Hsp22 negatively regulated cardiac injury in response to doxorubicin treatment through blocking TLR4/NLRP3 inflammasome activation (Lan et al., 2020). Combination of doxorubicin and trastuzumab, a targeted chemotherapy drug, significantly increased the incidence of cardiotoxicity (Mohan et al., 2018). Similarly, the TLR4/MyD88/NF-κB signaling and NLRP3 inflammasome pathway contributed to the cardiotoxicity of doxorubicin combined with trastuzumab (Maurea et al., 2020). To sum up, NLRP3 inflammasome participated in the initiation and progression of doxorubicin-mediated cardiotoxicity by inducing cardiomyocytes apoptosis, pyroptosis, and myocardium inflammatory reaction.

### Cutaneous Reaction

#### Carbamazepine-Induced Cutaneous Reaction

Most idiosyncratic drug reactions appear to be immune mediated, especially cutaneous reactions (Uetrecht and Naisbitt, 2013). Among them, Stevens-Johnson syndrome (SJS) and toxic epidermal necrolysis (TEN) are life-threatening with high morbidity and mortality (Seminario-Vidal et al., 2020). The main metabolite of carbamazepine (carbamazepine-10, 11-epoxide) promoted NLRP3 inflammasome activation in SJS/TEN keratinocytes in an AhR/MLKL/NLRP3/IL-1β-dependent pathway. Increased NLRP3 and IL-1β expressions were detected in serum and abnormal tissue of patients with SJS/TEN (Zhang C. et al., 2018). Activation of NLRP3 inflammasome aggravated CD8^+^ T-cell skin migration, and the increased IL-1β level was significantly correlated with Granulysin, soluble Fas ligand (sFasL), and C-X-C motif chemokine 10 (CXCL10) in patient serum and tissues. Thereby NLRP3 inflammasome contributed to the unbalanced inflammatory response in patients with SJS/TEN (Zhang C. et al., 2018).

#### Imiquimod-Induced Cutaneous Reaction

Imiquimod is a ligand for TLR7 and TLR8 as well as a potent immune activator in topical application. It has been reported that imiquimod caused a psoriasis-like disease or exacerbated lesions in patients with well-controlled psoriasis (Patel et al., 2011). In the mouse model, imiquimod increased expression of p-NF-κB and NLRP3 inflammasome activation in skin. Co-treatment with BAY 11-7082 (a NF-κB inhibitor) attenuated psoriasis, along with reduced expressions of p-NF-κB, NLRP3, IL-1β, TNF-α, and IL-6 (Irrera et al., 2017). However, NLRP3 knockout only partly reduced psoriasis-like dermatitis in mice (Irrera et al., 2017). Though NF-κB is a powerful activation regulator of NLRP3, the contribution of NLRP3 inflammasome to imiquimod-induced psoriasis remains controversial considering the broad ant-inflammatory activity of BAY 11-7082 (Juliana et al., 2010).

#### Nevirapine-Induced Cutaneous Reaction

The use of non-nucleoside reverse transcriptase inhibitor nevirapine is associated with a relatively high incidence of serious idiosyncratic drug reactions, especially skin rashes and hepatotoxicity (Pollard et al., 1998). It has been reported that death-associated protein kinase 1 (DAPK 1) is required for the caspase-1 activation and full IL-1β production through directly binding to NLRP3 (Chuang et al., 2011). Microarray studies of rat skin demonstrated that gene expression of DAPK significantly increased (3.37 fold) in 12-OH-nevirapine (a metabolite of nevirapine) treated rats (Zhang et al., 2013). As DAPK 1 is implicated in early autophagy events, apoptosis, and cell damage (Chuang et al., 2011), it implies that nevirapine induced cutaneous abnormality by DAPK 1-mediated NLRP3 inflammasome activation and cell injury.

#### Telaprevir and Dimethyl Fumarate-Induced Cutaneous Reaction

Telaprevir/boceprevir and dimethyl fumarate/ethacrynic acid are two pairs of similar chemically reactive drugs. The drug skin reactions caused by telaprevir and dimethyl fumarate activated IL-1β secretion in THP-1 human peripheral blood monocyte cells, while boceprevir and ethacrynic acid did not (Weston and Uetrecht, 2014). It can be inferred that activation of NLRP3 inflammasomes may be a possible biomarker in drug-caused idiosyncratic skin reactions.

### Gastrointestinal Reaction

#### 5-Fluorouracil-Induced Gastrointestinal Reaction

The fluoropyrimidine antimetabolite 5-fluorouracil is one of the most common anticancer agents, and it induced intestinal mucositis in 40–80% of patients, which causes adverse digestive effect such as diarrhea and dehydration (Sonis, 2004). 5-Fluorouracil exposure resulted in small intestinal mucositis with diarrhea and body weight loss, accompanied by elevated NLRP3, cleaved caspase-1, and IL-1β in the lamina propria and damaged epithelial cells in the mouse model (Nakata et al., 2019). Genetic depletion or pharmacological inhibition of NLRP3 and caspase-1 attenuated 5-fluorouracil-induced mucositis. Small intestinal mucositis was aggravated by exogenous IL-1β but ameliorated by IL-1β antibody treatment (Nakata et al., 2019). Microscopically, 5-fluorouracil-induced intestinal mucositis is characterized by villous length reduction and structural changes with inflammation (Justino et al., 2015). It has been suggested that NLRP3 inflammasome activation exacerbated 5-fluorouracil-induced small intestinal mucositis via promoting IL-1β maturation (Nakata et al., 2019).

#### Cyclophosphamide-Induced Gastrointestinal Reaction

Cyclophosphamide is used in chemotherapy and is broken down to acrolein that stores in the urine before excretion, in which it can interact with the bladder wall and trigger a massive hemorrhagic cystitis (Anton, 2002). Immunohistochemistry showed that NLRP3 was expressed in the bladder and localized predominantly in the urothelial. The caspase-1 activity and IL-1α secretion were enhanced after 4 h of cyclophosphamide exposure, while the inflammation and bladder dysfunction were triggered after 24 h (Hughes et al., 2014). Inhibiting NLRP3 blocked inflammasome activity, reduced inflammation, and reversed bladder dysfunction, though there was no change in the IL-1β level (Hughes et al., 2014).

#### Methamphetamine-Induced Gastrointestinal Reaction

Methamphetamine is a crucial component of the popular illegal drug “ICE.” Except for its neurotoxic effects, methamphetamine, orally taken, has also caused severe inflammatory injury of the intestine in several clinical cases (McKelvie and Gercek, 2017). Methamphetamine resulted in increased cell apoptosis and the levels of NF-κB, IL-6, TNF-α, and INF-γ *in vitro* and vivo, as well as a decrease in transepithelial electrical resistance, which could be ameliorated by MCC950 (Zhao et al., 2019). As an important upstream regulator of NLRP3 inflammasome, whether NF-κB promoted the NLRP3 inflammasome activation in methamphetamine-induced intestinal inflammatory injury needs further verification (Zhao et al., 2019).

### Neurotoxicity

#### Bortezomib-Induced Neurotoxicity

The clinical use of bortezomib often led to chronic painful neuropathy mainly presenting as spontaneous pain and mechanical hypersensitivity in cancer patients, caused by dose reduction or discontinuation during chemotherapy (Broyl et al., 2012). The upregulation of NLRP3 in dorsal root ganglion mediated by signal transducer and activator of transcription-3 (STAT3)-dependent histone acetylation is critically involved in bortezomib-induced mechanical allodynia (Liu et al., 2018).

#### Cyclophosphamide-Induced Neurotoxicity

Besides hemorrhagic cystitis, inflammation and depression induced by cyclophosphamide was also dependent on the activation of NLRP3 inflammasome (Hirshman et al., 2020). An acute insult of cyclophosphamide in the bladder can trigger significant neuroinflammation in the hippocampus, which brought about symptoms of depression in rats. Thus, NLRP3 inflammasome may act as a bridge between benign bladder disorders and mood disorders induced by cyclophosphamide (Hirshman et al., 2020).

#### Morphine-Induced Neurotoxicity

Morphine is an opioid used for pain management. Paradoxically, a short course of morphine after nerve injury doubles the duration of neuropathic pain in rats (Grace et al., 2016). Grace et al. (2016) demonstrated that morphine induced the formation and activation of NLRP3 inflammasome and prolonged neuropathic pain in spinal microglia. Inhibiting spinal cord NLRP3 inflammasome signaling by genetic and pharmacological interventions could permanently reset amplified pain to base levels.

#### Paclitaxel-Induced Neurotoxicity

Paclitaxel, a commonly used drug for cancer chemotherapy, frequently causes peripheral neuropathic pain (Park et al., 2011). Paclitaxel elicited mitochondria damage, ROS production, and activation of NLRP3 inflammasome in L4-6 dorsal root ganglia and sciatic nerve of rats, which led to dorsal root ganglia and sciatic nerve pain (Jia et al., 2017). *In vitro*, paclitaxel also increased the number of damaged mitochondria and mtROS production in the rat alveolar macrophage cell line NR8383. The elicited mitochondria damage and ROS production promoted the activation of NLRP3 inflammasome in peripheral nerve, which contributed to paclitaxel-induced neuropathic pain (Jia et al., 2017).

### Lung Injury

#### Aspirin-Induced Lung Injury

Aspirin-induced asthma is a common clinical symptom of aspirin hypersensitivity, and this acute reaction is elicited through cyclooxygenases inhibition by non-steroidal anti-inflammatory drugs (Szczeklik and Sanak, 2006). Single nucleotide polymorphisms (SNPs) analysis indicated that the NLRP3 SNPs might play a significant role in the development of aspirin-induced asthma in a gain-of-function manner (Hitomi et al., 2009). Further research on the NLRP3 inflammasome will contribute to the development of novel diagnostic and therapeutic methods for aspirin-induced asthma (Hitomi et al., 2009).

#### Bleomycin-Induced Lung Injury

The anticancer agent bleomycin can cause DNA breakdown and ROS generation, which is cytotoxic and results in remodeling of lung architecture and loss of pulmonary function (Della Latta et al., 2015). In A549 and RLE-6TN cells, HIF-1α modulated the NLRP3 inflammasome activation and IL-1β secretion through NF-κB signaling in bleomycin-induced cell toxicity (Huang et al., 2019).

#### Pravastatin-Induced Lung Injury

Though statins, the 3-hydroxy-3-methylglutaryl-coenzyme A reductase inhibitors, are common medications for hypercholesterolemia, observations suggested that they could cause various types of interstitial lung diseases characterized by diffuse pulmonary parenchyma, alveolar inflammation, and interstitial fibrosis (Fernandez et al., 2008). In the mouse model, pravastatin pretreatment exacerbated bleomycin-induced lung fibrosis and the activation of NLRP3 inflammasome, which was dependent on up-regulation of mtROS. Moreover, pravastatin enhanced the secretions of IL-1β and IL-18 in macrophages stimulated with LPS and ATP. These results indicated that activation of NLRP3 inflammasome induced by pravastatin augmented lung inflammation and ultimately exacerbated the development of bleomycin-induced interstitial lung diseases (Xu et al., 2012).

## Phytochemicals That Suppress Drug-Induced Toxicity by Inhibiting NLRP3 Inflammasome

In the previous section, we systematically summarized different organ toxicities caused by diverse drugs, in which the NLRP3 inflammasome played an important role. A further focus is the application of our understanding for the NLRP3 inflammasome activation on assessing their therapeutic potential. Documented data demonstrated that several phytochemicals could reduce different drug-induced organ damage *in vitro* cell models and *in vivo* animal experiments by directly targeting NLRP3 inflammasome.

### Phytochemicals That Treat Drug-Induced Liver Injury

As mentioned previously, acetaminophen hepatotoxicity has been studied extensively. Abundant experimental research also highlighted the protective effects of natural products. Daphnetin (a natural coumarin derivative) alleviated acetaminophen-induced hepatotoxicity by inhibition of TXNIP/NLRP3 activation, which was strongly dependent on the upregulation of Nrf2/Trx-1 axis (Lv et al., 2020). The natural alkaloid-sinomenine treatment dose-dependently attenuated acetaminophen-induced NLRP3 inflammasome activation and cytokines secreted via TGF-β/Smad pathway *in vitro* and vivo (Chen H. et al., 2020). Kaempferol, a flavonoid compound derived from the medicinal and edible plant of *Penthorum chinense* Pursh, has been reported to exert a profound anti-inflammatory and antioxidant activity (Alam et al., 2020). Recently, data revealed that kaempferol pretreatment significantly suppressed the expression of NLRP3 and the secretion of IL-1β, TNF-α, and IL-6. Kaempferol also reduced the AST and ALT levels, relieved hepatocellular damage and apoptosis. Kaempferol could protect hepatocytes from acetaminophen hepatotoxicity through the inhibition of the HMGB1/TLR4/NF-κB signaling pathway (Du et al., 2020). Similarly. Allicin (an active sulfur species isolated from garlic) also protected the mouse liver from acetaminophen-induced necrosis, apoptosis, and hepatocellular degeneration via inhibiting the hepatic NLRP3 inflammasome activation and IL-1β secretion and increasing Ki-67 level (Samra et al., 2020). Liver regeneration has drawn a lot of attention in the study of DILI (Bhushan and Apte, 2019; Clemens et al., 2019). A recent study showed that baicalin promoted liver regeneration via inducing Nrf2 accumulation in cytoplasm, thus causing NLRP3 inflammasome activation after acetaminophen-induced acute liver injury in mice (Shi et al., 2020). It seems that the NLRP3 inflammasome activation partly acted as a compensatory regulation in the process of acetaminophen hepatotoxicity.

Quercetin has multiple pharmacological properties and is regarded as a potential protective agent against organ injuries (Salehi et al., 2020). Quercetin pretreatment reduced the level of aminopherases and improved the morphological changes in rat liver and L02 cells administered with isoniazid. *In vitro* study indicated that quercetin exhibited protective effects against isoniazid-induced liver damage via inhibiting the activation of NLRP3 inflammasome and apoptosis in a SIRT-dependent manner (Zhang Y. M. et al., 2020).

### Phytochemicals That Treat Drug-Induced Kidney Injury

Pyrroloquinoline quinone is a new type of water-soluble anionic redox compound with anti-oxidative, anti-inflammatory, hepatoprotective, and neuroprotective activities (Jiang et al., 2020; Zhang H. Y. et al., 2020). Compared to cyclophosphamide group, pyrroloquinoline quinone co-treatment significantly decreased the serum levels of creatinine and urea in mice, as well as the levels of IL-1β, IL-6, TNF-α, and malonaldehyde (Lin X. et al., 2020). Moreover, pyrroloquinoline quinone inhibited the NLRP3 inflammatory pathway, as indicated by the reduced expressions of NLRP3, ASC, and caspase-1 (Zhang H. Y. et al., 2020).

Ferulic acid is a hydroxycinnamic acid found in plant cell walls as side chains of arabinoxylans and has been recently reported to protect against AKI induced by gentamicin through upregulation of PPAR-γ (El-Ashmawy et al., 2018). Chicoric acid is a dicaffeyltartaric acid and a natural compound that occurs in a variety of plant species used in folk medicine, such as Cichorium intybus L. and Echinacea purpurea (Lee and Scagel, 2013). Both ferulic acid (Mahmoud et al., 2019) and chicoric acid (Abd El-Twab et al., 2019) were proven to be able to inhibit ROS-induced activation of NF-κB/NLRP3 inflammasome signaling by activating Nrf2/ARE/HO-1 axis, thus ultimately preventing methotrexate-induced kidney injury.

### Phytochemicals That Treat Doxorubicin-Induced Cardiotoxicity

Apoptosis, pyroptosis, and defective autophagy are believed to contribute to doxorubicin-induced cardiotoxicity. Calycosin is the major active component in Radix astragali, which has emerged as a highly valued herb to treat cardiovascular and renal disease (Gao et al., 2014). Calycosin could increase H9c2 cells’ viability and reduce apoptosis induced by doxorubicin in mice heart via decreasing the activation of Sirt1/NLRP3 pathway (Zhai et al., 2020). Similarly, dihydromyricetin protected against doxorubicin-induced cardiotoxicity by inhibiting NLRP3 inflammasome activation via stimulation of the Sirt1 pathway in rats and H9c2 cells (Sun et al., 2020). *In vivo*, impaired cardiac functions induced by doxorubicin were overtly reconciled by 100 mg/kg curcumin (a monomer substance derived from turmeric) in mice through regulation of autophagy and pyroptosis in a mTOR-dependent manner (Yu W. et al., 2020). Honokiol, an effective ingredient extracted from the bark of Magnolia officinalis, was also found to be effective in protecting cardiomyocytes against doxorubicin-stimulated senescence mediated via the inhibition of TXNIP expression and the subsequent suppression of the NLRP3 inflammasome (Huang et al., 2020).

## Perspectives and Conclusion

NLRP3 inflammasome has consistently been shown to play a vital role in drug-induced toxicity through inducing different forms of cell death and inflammatory responses in the initial stage of toxicity (Zhou et al., 2011; Subramanian et al., 2013). Cell death (apoptosis, necrosis, autophagy, and pyroptosis) and irreversible tissue damage are severe symptoms of drug toxicity. Moreover, a later stage of injury mediated by the secreted inflammatory factors (mainly IL-1β) and recruitment of inflammatory cells further contributed to toxicity (Baroja-Mazo et al., 2014). In our review, ROS overproduction and NF-κB transcription were responsible for the activation of NLRP3 inflammasome in many drug-induced tissue injuries. The mitochondria damage, DAMPs, and mitophagy also contributed to the NLRP3 inflammasome activation in DILI, while autophagy and microRNAs are important stimulants of NLRP3 inflammasome in the progress of nephrotoxicity. Considering the diversity of NLRP3 inflammasome stimuli, it seems that NLRP3 may sense a common triggering pathway induced by multiple intracellular processes. Most drugs promoted the secretion of IL-1β, TNF-α, and IL-6 in the liver, kidney, skin, and gastrointestinal tract. In the heart, NLRP3 inflammasome activation induced by doxorubicin and aconitine mainly led to the maturity and release of IL-1β. And research about inflammatory production in the nervous system and lung need further exploration. Though IL-18 is a classical downstream cytokine of NLRP3 inflammasome, little research has shown elevated IL-18 level in drug-induced toxicity. Further intensive investigations will help to interpret the specific regulatory mechanisms of NLRP3 inflammasome in organ toxicity induced by different drugs in the future.

Fruitful progress has been made to inhibit NLRP3 inflammasome by the blockage of NLRP3, NF-κB, P2X7R, caspase-1, TLRs, IL-1β, and IL-18 (Peiro et al., 2017; Su et al., 2020). So far, MCC950 serves as the most effective inhibitor of NLRP3 with a cellular IC_50_ of 8 nM and is used to investigate NLRP3-dependent pathology in pre-clinical models (Coll et al., 2015). Therapeutic strategies targeting NLRP3 inflammasome have generated great interest too (Mangan et al., 2018), especially natural products such as kaempferol, quercetin, and chicoric acid, which have shown beneficial effects on drug-induced toxicities through direct or indirect NLRP3 inflammasome inhibition (Abd El-Twab et al., 2019; Du et al., 2020; Zhang Y. M. et al., 2020). Oridonin is the major active ingredient of the traditional Chinese medicinal herb Rabdosia rubescens and has strong anti-inflammatory activity. It is noteworthy that oridonin forms a covalent bond with the cysteine 279 of NLRP3 in NACHT domain to block the interaction between NLRP3 and NEK7, thereby inhibiting the assembly and activation of NLRP3 inflammasome (He et al., 2018). Moreover, oridonin has both preventive and therapeutic effects on mouse models of peritonitis, gouty arthritis, and type 2 diabetes (He et al., 2018). Therefore, it is highly promising that phytochemicals of high safety and extensive origin can be used to attenuate drug-induced toxicity by targeting the over-activation of NLRP3 inflammasome. Recently, emerging evidence suggests that NLRP3 may acts as an oncogene in many tumors (Bruchard et al., 2013; Karki et al., 2017). NLRP3 inflammasome and IL-1β production promote the infiltration of myeloid cells, providing an inflammatory microenvironment for breast cancer progression (Ershaid et al., 2019). Moreover, NLRP3 inflammasome in fibroblasts is further linked with tumor progression and metastasis (Chen Q. et al., 2020). Considering the vital role of NLRP3 inflammasome in doxorubicin-induced cardiotoxicity, we speculate that targeting the NLRP3 inflammasome can attenuate the cardiotoxic effects of doxorubicin while enhancing its anti-cancer activity.

Several paradoxical conclusions exist on the same drug in different studies, which may be due to different methods, cell types, animal models, and dosage regimens. In addition, many pathway responses to different inflammasomes are interrelated and overlapping (Fernandes et al., 2020), and underappreciated inflammasomes can also drive IL-1β and IL-18 secretion in response to stimuli (Broz and Dixit, 2016). Technical limitations, such as the absence of conditional NLRP3 gene knockout and the lack of *in vivo* validation for *in vitro* results, also lead to conflicting conclusions.

In this review, we summarize and discuss the role of NLRP3 inflammasome in the drug-induced diverse toxicities. NLRP3 inflammasome appears to be a promising target for treating drug-induced toxicity. Further investigations on the underlying molecular mechanisms and therapy exploration will advance our understanding of NLRP3 inflammasome, and eventually improve the safety of medicine use.

## Author Contributions

SW and WM wrote the manuscript. BZ and WL revised the manuscript. All authors read and approved the final version of the manuscript for publication.

## Conflict of Interest

The authors declare that the research was conducted in the absence of any commercial or financial relationships that could be construed as a potential conflict of interest.
