# Supplementary material for: NLRP3 Inflammasome: A Promising Therapeutic Target for Drug-Induced Toxicity
Source: Front Cell Dev Biol. 2021 Apr 12;9:634607. doi: 10.3389/fcell.2021.634607 (PMC8072389; doi:10.3389/fcell.2021.634607)
Supplement: Supplementary file 1 [file Table_1.DOCX]

| \| **Table 1.** Role of NLRP3 inflammasome activation in drug-induced toxicity \| \| \| \| \| \| --- \| --- \| --- \| --- \| --- \| \| Organ \| Drug \| Patient/Animal/Cell types \| Mechanisms \| references \| \| Liver \| Acetaminophen \| C57BL/6 mice \| NLRP3 inflammasome↑-IL-1β↑ \| (Williams et al., 2010; Williams et al., 2011; Samra et al., 2020) \| \| C57BL/6 mice, HepG2 cells \| TXNIP↑-NLRP3 inflammasome↑-IL-1β↑ \| (Lv et al., 2020) \| \| Mice, BRL-3A cells \| NLRP3 inflammasome↑-IL-1β↑ \| (Chen et al., 2020) \| \| C57BL/6 mice, THP-1 cells,  primary mouse LSECs \| DAMPs↑-NLRP3↑-IL-1β↑;  Apoptotic DNA↑-TLR9↑-IL-1β↑ \| (Imaeda et al., 2009) \| \| C57BL/6 mice \| Mitochondria NAD and ATP↑-P2X7↑-NLRP3↑-IL-1β↑ \| (Sohail et al., 2010; Hoque et al., 2012) \| \| C57BL/6 mice \| Mitophagy↑-NLRP3 inflammasome↓-inflammatory response↓ \| (Shan et al., 2019) \| \| Hepatocytes isolated from male SD rats \| ER stress↑-NLRP3↑-IL-1β↑ \| (Kim and Park, 2018) \| \| BALB/c mice,  primary mouse hepatocytes \| IL-1Ra↑-NF-κB↓-P450 enzyme expression↑ \| (Ishibe et al., 2009) \| \| C57BL/6 mice \| HMGB1/TLR4/NF-κB or NLRP3↑- IL-1β↑, IL-6↑, TNF-α↑ \| 179 \| \| Azathioprine \| BALB/c mice \| ROS↑-oxidative stress↑-hepatocyte necrosis↑-HMGB1 and S100A8↑-NALP3↑-IL-1β↑ \| (Matsuo et al., 2014) \| \| Busulfan and Cyclophosphaide \| C57BL/6 mice, BALB/c mice \| NLRP3↑-caspase-1↑, ATP↑, HMGB1↑ \| (Qiao et al., 2015) \| \| Carbamazepine \| C57BL/6 mice, BMDMs,  THP-1 cells, HEK-293T cells \| NLRP3 inflammasome↑ \| (Wang et al., 2019b) \| \| Epimedii Folium \| C57BL/6 mice, bone marrow cells \| mtROS↑-NLRP3↑-caspase-1↑-IL-1β↑ \| (Wang et al., 2020) \| \| Epimedii Folium and Psoraleae Fructus \| SD rats \| NLRP3↑-IL-1β↑, IL-6↑, TNF-α↑-IFN-γ↑ \| (Gao et al., 2020) \| \| Psoralidin \| C57BL/6 mice; BMDMs \| NLRP3↑-caspase-1↑-IL-1β↑ \| (Wang et al., 2021) \| \| Isoniazid \| SD rats \| SirT1↓-NLRP3↑-caspase-1↑ \| (Zhang et al., 2020) \| \| Phenytoin \| C57BL/6J mice \| p450-dependent metabolic↑-DAMPs↑-NLRP3↑-IL-1β↑ \| (Sasaki et al., 2013) \| \| Liver \| Polygonum multiflorum \| HepG2 cells, THP-1 derived macrophages \| NLRP3 inflammasome↑-IL-1β↑ \| (PAN Yun-zheng et al., 2021) \| \| Triptolide \| C57BL/6 mice \| Liver damage↑-TLR4/Myd88/NF-κB-NLRP3↑-caspase-1↑-IL-1β↑ \| (Yuan et al., 2019) \| \| Kidney \| Aristolochic Acid \| BALB/c mice, HK-2 cells \| NLRP3↑-caspase-1↑-IL-1β↑ \| (Wang et al., 2019a) \| \| HKC cells, HK-2 cells \| NLRP3 inflammasome↑-IL-1β and IL-18↑,  α-SMA↑, E-cadherin↓ \| (Yu et al., 2020a) \| \| Cisplatin \| CD1 mice, HEK cells \| HSP70↑-NLRP3 inflammasome↑-IL-1β and IL-18↑ \| (Ullah et al., 2020) \| \| C57BL/6 mice, HK-2 cells \| Mitochondria ROS↑-NLRP3↑-caspase-1↑-IL-1β↑-apoptosis↑ \| (Yang et al., 2020) \| \| MTECs, HK-2 cells \| NOX4↑-ROS↑-NLRP3↑-apoptosis↑ \| (Ma et al., 2019) \| \| C57BL/6 mice \| Caspase-1↑-apoptosis and acute tubular necrosis↑ \| (Faubel et al., 2004) \| \| C57BL/6 mice,  RAW 264.7 cells and TECs \| NLRP1 inflammasome↑-caspase-5↑, a trend of NLRP3↑ \| (Kim et al., 2013) \| \| SD rats \| NLRP3 inflammasome↑-IL-1β and IL-18↑ \| (Qu et al., 2019a) \| \| SD rats \| α-SMA↑, cleaved **c**aspase 3↑, NLRP3↑ \| (Li et al., 2020) \| \| SD rats \| Autophagy↓-mitochondria damage↑-NLRP3 inflammasome↑ \| (Qu et al., 2019b) \| \| C57BL/6 mice, proximal tubules \| Caspase-1↑-IL-1α and IL-1β↑-necrosis↑ \| (Lee et al., 2015) \| \| C57BL/6 mice \| P2X7R↑-NLRP3 inflammasome↑,oxidative stress↑,caspase-3↑ \| (Zhang et al., 2014) \| \| Cyclophosphamide \| ICR mice \| Inflammation↑- NLRP3↑-caspase-1↑ \| (Lin et al., 2020b) \| \| Iohexol \| SD rats \| NLRP3 inflammasome↑-HIF1α↓-BNIP3↓- mitophagy↓ \| (Lin et al., 2020a) \| \| C56BL/6J mice, HK-2 cells \| mitochondria damage↑-PINK1-Parkin/mitophagy↑-mtROS↑- NLRP3 inflammasome↑-IL-1β↑-apoptosis↑ \| (Lin et al., 2019) \| \| Miniature pig \| miRNA-30c↑-NLRP3↓-caspase-1↓-IL-1β↓ \| (Xu et al., 2020) \| \| C57BL/6N mice,  primary human and mouse renal TECs \| Caspases 4/5/↑-IL-1β↑-pyroptosis↑ \| (Zhang et al., 2018b) \| \| C57/BL6 mice,  primary human proximal TECs \| Dipeptidase-1-NLRP3 inflammasome↑-IL-1β↑ \| (Lau et al., 2018) \| \| Iopromide \| SD rats, NRK-52e cells \| ROS↑-S100A8/A9/TLR4/NLRP3/apoptotic↑ \| (Tan et al., 2017) \| \| Ioversol \| SD rats \| LncRNA XIST↑- miR-133a-3p↓-NLRP3↑-caspase-1↑ \| (Liu et al., 2021) \| \| Isopaque and **o**mnipaqu \| C57BL/6 mice, HK-2 cells \| NLRP3↑-ASC↑-IL-1β and IL-18↑-apoptosis↑ \| (Shen et al., 2016) \| \| Methotrexate \| Wistar rats \| ROS↑-NF-κB↑-NLRP3↑-caspase-1↑-IL-1β↑-apoptosis↑ \| (Abd El-Twab et al., 2019; Mahmoud et al., 2019) \| \| Heart \| Aconitine \| SD rats, H9c2 cells \| BNIP3↓-mitophagy↓-NLRP3 inflammasome↑-apoptosis↑ \| (Peng et al., 2020) \| \| Doxorubicin \| C57BL/6J mice,  murine peritoneal macrophages \| NLRP3↑-IL-10↑-susceptibility↑ \| (Kobayashi et al., 2016) \| \| SD rats; H9c2 cells \| NLRP3↑-caspase-1↑-IL-1β and IL-18↑ \| (Sun et al., 2020) \| \| C57BL/6J mice \| ER stress↑-necrosis↑-DAMP/TLR4/NLRP3 inflammasome↑-pyroptosis↑ \| (Singla et al., 2019) \| \| Wistar rats, H9c2 cells,  primary neonatal rat cardiomyocytes \| lncRNA TINCR↑-IGF2BP1↑-NLRP3 inflammasome/ GSDMD/cardiomyocyte pyroptosis \| (Meng et al., 2019) \| \| C57BL/6J mice, H9c2 cells \| NOX1 and NOX4-Drp1-ROS- NLRP3↑-caspase-1↑-IL-1β↑ \| (Zeng et al., 2020) \| \| C57BL/6 mice, H9c2 cells \| ROS↑-NLRP3↑-caspase-1↑-Bax/Bcl-2↑-apoptosis↑, IL-1β↑ \| (Wei et al.) \| \| H9c2 cells \| TXNIP↑-NLRP3↑-caspase-1↑-IL-1β↑-inflammation and senescence↑ \| (Huang et al., 2020) \| \| C57BL/6J mice,  primary mouse cardiac fibroblast \| TMAO-NLRP3 inflammasome↑-cardiac fibrosis↑ \| (Li et al., 2019) \| \| C57BL/6 mice, H9c2 cells \| HSP22↑-TLR4/NLRP3↓-apoptosis↓ \| (Lan et al., 2020) \| \| Kunming mice, H9c2 cells \| Sirt1↓-TXNIP↑-NLRP3↑-caspase-1↑-IL-1β↑ \| (Zhai et al., 2020) \| \| Kunming mice, H9c2 cells \| PI3K/Akt/mTOR↑-NLRP3↑-caspase-1↑-IL-18↑ \| (Yu et al., 2020b) \| \| Doxorubicin  and trastuzumab \| HL-1 adult cardiomyocytes \| TLR4/MYD88/NF-κB/NLRP3 inflammasome↑ \| (Maurea et al., 2020) \| \| Skin \| Carbamazepine \| Serum and tissue of patients with SJS/TEN \| AhR↑-MLKL↑-NLRP3↑-IL-1β↑-CD8+T-cell skin migration \| (Zhang et al., 2018a) \| \| Imiquimod \| C57BL/6 mice \| NLRP3↑-IL-1β↑ \| (Irrera et al., 2017) \| \| Nevirapine \| Brown Norway rats \| TRIM63 and DAPK1↑-NLRP3 inflammasomes↑ \| (Zhang et al., 2013) \| \| Dimethyl fumarate \| THP-1 cells \| IL-1β↑ \| (Weston and Uetrecht, 2014) \| \| Intestine \| 5-Fluorouracil \| C57BL/6 mice \| NLRP3↑-caspase-1↑- IL-1β and IL-18↑ \| (Nakata et al., 2019) \| \| Methamphetamine \| C57BL/6 mice, IEC-6 \| NLRP3 inflammasome↑- IL-1β and IL-18↑-apoptosis↑ \| (Zhao et al., 2019) \| \| Bladder \| Cyclophosphamide \| SD rats \| NLRP3↑-IL-1β↑ \| (Hughes et al., 2014) \| \| Nerves \| Bortezomib \| SD rats \| STAT3↑-histone acetylation↑-NLRP3↑ \| (Liu et al., 2018) \| \| Cyclophosphamide \| SD rats \| NLRP3↑-ASC↑- IL-1β and IL-18↑ \| (Hirshman et al., 2020) \| \| Morphine \| SD rats \| TLR4/NF-κB↑-NLRP3↑-caspase-1↑-IL-1β↑ \| (Grace et al., 2016) \| \| Paclitaxel \| SD rats \| Mitochondria damage↑-ROS↑-NLRP3↑-caspase-1↑-IL-1β↑ \| (Jia et al., 2017) \| \| Lung \| Aspirin \| Patients \| SNPs with genomic DNA \| (Hitomi et al., 2009) \| \| Bleomycin \| A549 cells, rat type ii alveolar cells \| HIF-1α↑-NF-κB↑-NLRP3 inflammasome↑-IL-1β↑ \| (Huang et al., 2019) \| \| Bleomycin and statins \| Patients, macrophages and BMDMs, C57B/L6 mice \| mtROS↑-NLRP3↑-caspase-1↑- IL-1β and IL-18↑ \| (Xu et al., 2012) \| |
| --- | --- | --- | --- | --- | --- | --- | --- | --- | --- | --- | --- | --- | --- | --- | --- | --- | --- | --- | --- | --- | --- | --- | --- | --- | --- | --- | --- | --- | --- | --- | --- | --- | --- | --- | --- | --- | --- | --- | --- | --- | --- | --- | --- | --- | --- | --- | --- | --- | --- | --- | --- | --- | --- | --- | --- | --- | --- | --- | --- | --- | --- | --- | --- | --- | --- | --- | --- | --- | --- | --- | --- | --- | --- | --- | --- | --- | --- | --- | --- | --- | --- | --- | --- | --- | --- | --- | --- | --- | --- | --- | --- | --- | --- | --- | --- | --- | --- | --- | --- | --- | --- | --- | --- | --- | --- | --- | --- | --- | --- | --- | --- | --- | --- | --- | --- | --- | --- | --- | --- | --- | --- | --- | --- | --- | --- | --- | --- | --- | --- | --- | --- | --- | --- | --- | --- | --- | --- | --- | --- | --- | --- | --- | --- | --- | --- | --- | --- | --- | --- | --- | --- | --- | --- | --- | --- | --- | --- | --- | --- | --- | --- | --- | --- | --- | --- | --- | --- | --- | --- | --- | --- | --- | --- | --- | --- | --- | --- | --- | --- | --- | --- | --- | --- | --- | --- | --- | --- | --- | --- | --- | --- | --- | --- | --- | --- | --- | --- | --- | --- | --- | --- | --- | --- | --- | --- | --- | --- | --- | --- | --- | --- | --- | --- | --- | --- | --- | --- | --- | --- | --- | --- | --- | --- | --- | --- | --- | --- | --- | --- | --- | --- | --- | --- | --- | --- | --- | --- | --- | --- | --- | --- | --- | --- | --- | --- | --- | --- | --- | --- | --- | --- | --- | --- | --- | --- | --- | --- | --- | --- |

BMDMs, bone marrow derived macrophages; BNIP3, BCL2/adenovirus E1B interacting protein 3; CTRP6, C1q/tumor necrosis factor related protein 6; DAMPs, damage- associated molecular patterns; DAPK1, death-associated protein kinase 1; ER stress, endoplasmic reticulum stress; HIF-1α, hypoxia inducible factor 1, alpha subunit ; HMGB1, high mobility group protein; HSP, heat shock protein; IEC-6, intestinal epithelial cell line 6. IGF2BP1, insulin like growth factor 2 mRNA binding protein 1; IL-1R1, IL-1 receptor type 1; lncRNA TINCR, terminal differentiation-induced non-coding RNA; LncRNA XIST, long non-coding RNA X-inactive specific transcript; LSECs, liver sinusoidal endothelial cells; LncRNA XIST, long non-coding RNA X-inactive specific transcript; MTECs, mouse tubular epithelial cells; MyD88, myeloid differentiation factor 88; NAD; nicotinamide adenine dinucleotide; NF-κB, nuclear factor-κB; NLRP3 inflammasome, including NLRP3, ASC and caspase-1; NOX1/4, nicotinamide adenine dinucleotide phosphate oxidase 1/4; PAMPs, pathogen- associated molecular patterns; SNPs, Single nucleotide polymorphisms; P2X7, P2X purinoreceptor 7; TECs, tubular epithelial cells; TNF-α, tumor necrosis factor-alpha; TLR, toll-like receptor; TMAO, Trimethylamine N-oxide; TRIM63, Tripartite Motif-containing Protein 63.
